# Crosslinking Behavior of UV-Cured Polyorganosilazane as Polymer-Derived Ceramic Precursor in Ambient and Nitrogen Atmosphere

**DOI:** 10.3390/polym13152424

**Published:** 2021-07-23

**Authors:** Afnan Qazzazie-Hauser, Kirsten Honnef, Thomas Hanemann

**Affiliations:** 1 Laboratory for Materials Processing, Department of Microsystems Engineering, University of Freiburg, 79110 Freiburg, Germany; kirsten.honnef@imtek.uni-freiburg.de; 2 Institute for Applied Materials, Karlsruhe Institute of Technology (KIT), Hermann-von-Helmholtz-Platz 1, 76344 Eggenstein-Leopoldshafen, Germany

**Keywords:** polysilazane, crosslinking, UV-LED photopolymerization, preceramic polymers, FTIR spectroscopy

## Abstract

Polymer-derived ceramics (PDCs) based on silicon precursor represent an outstanding material for ceramic coatings thanks to their extraordinary versatile processibility. A promising example of a silicone precursor, polyorganosilazane (*Durazane 1800*), was studied concerning its crosslinking behavior by mixing it with three different photoinitiators, and curing it by two different UV-LED sources under both nitrogen and ambient atmosphere. The chemical conversion during polymerization and pyrolysis was monitored by FTIR spectroscopy. Pyrolysis was performed in a nitrogen atmosphere at 950 °C. The results demonstrate that polyorganosilazane can be cured by the energy-efficient UV-LED source at room temperature in nitrogen and ambient atmosphere. In nitrogen atmosphere, already common reactions for polysilazanes, including polyaddition of the vinyl group, dehydrogenation reactions, hydrosilylation, and transamination reaction, are responsible for crosslinking. Meanwhile, in ambient atmosphere, hydrolysis and polycondensation reactions occur next to the aforementioned reactions. In addition, the type of photoinitiator has an influence on the conversion of the reactive bonds and the chemical composition of the resulting ceramic. Furthermore, thermogravimetric analysis (TGA) was conducted in order to measure the ceramic yield of the cured samples as well as to study their decomposition. The ceramic yield was observed in the range of 72 to 78% depending on the composition and the curing atmosphere. The curing atmosphere significantly impacts the chemical composition of the resulting ceramics. Depending on the chosen atmosphere, either silicon carbonitride (SiCN) or a partially oxidized SiCN(O) can be produced.

## 1. Introduction

In recent years, polymer-derived ceramics (PDCs) based on silicon precursor such as polyorganosilazane have been used as precursors for ceramics [1], as they offer many advantageous properties, in particular excellent adhesion on numerous surfaces [2,3] as well as high thermal and chemical stability [4]. In addition, polysilazane serves as a component for the preparation of ceramic matrix composites produced by means of additive manufacturing [5,6]. Polyorganosilazane consists of an alternating silicon and nitrogen backbone and is produced on a large scale via ammonolysis of dichlorosilane (R_2_SiCl_2_) [7,8]. Like polymers, PDCs benefit from the extraordinary versatility of processing when compared with bulk ceramics. After processing, it is necessary to perform crosslinking followed by pyrolysis, leading to the desired ceramic. The crosslinking can be conducted thermally by adding a free radical initiator such as peroxides [9,10,11], catalysts [12], or photochemically [13,14,15], to form an infusible network. Another well-established method for the crosslinking of polysilazane and other PDCs is the thiol-ene click chemistry, which is also induced by means of UV light [16,17,18]. Thereby, the polymerization can occur by the addition of the thiol group to the vinyl group of polysilazane, which modifies the compound completely. Owing to the step-growth polymerization, the gelation happens when a high amount of functional groups has reacted [16].

UV-curing is a well-known fast and low-cost method for the fabrication of polymers [19,20] and can be perfomed at room temperature. Preliminary, UV-curing of polysilazane has been realized before by applying photoinitiators absorbing in the UV-C region [21,22]. Typically, the UV light source for this range is an environmentally harmful mercury-vapor lamp, as it delivers light in the 280–450 nm range, matching the absorption of the photoinitatiors [23]. In other works, polysilazane has been synthetically modified by mixing it with acrylates, resulting in a photocurable resin, which was cured by digital light processing [6,14]. The photoinitiator 2,2-dimethoxy-2-phenyl acetophenone, which has its absorbance maximum between 310 and 390 nm [24], was used by the authors Hu et al. [25] to cure polysilazane into free standing specimen (138 and 38 µm) followed by thermal deep curing. Another scientist used the same photoinitiator for the fabrication of SiCN MEMS [26]. In comparison with this work, the authors focus on the characterization of the material properties after the pyrolysis process and less on the crosslinking behavior of polysilazane.

In this work, the UV-curing approach was adopted, replacing the mercury-vapor lamp by the energy-efficient UV-LED source [20], to start the crosslinking reaction by free-radical polymerization.

The material presented here is a low-viscous and colorless polyorganosilazane, which has excellent adhesion properties to most surfaces. Therefore, it is used as coating material processed by spin- or dip-coating [3,27]. The solubility in most organic solvents and the resulting tailoring in viscosity make the material attractive for usage as binding material for filler-loaded preceramic polymer [28,29]. In inert atmosphere, the crosslinked network of polysilazane can be pyrolyzed into amorphous silicon carbonitride (SiCN) at a temperature of 800 °C, whereas at temperatures higher than 1400 °C, it crystallizes and phase separates into silicon nitride and silicon carbide [1,30,31].

The aim of this work is to investigate the crosslinking behavior of polyorganosilazane cured by ultraviolet light emitting diode (UV-LED) sources inducing the free-radical polymerization of different photoinitiators. The photoinitiators used are benzophenone derivate: dibenzosuberone (DBS), 4-(dimethylamino) benzophenone (DMABP), and isopropylthioxanthone (ITX). The crosslinking behavior of the mixtures was analyzed in detail before and after curing by FTIR spectroscopy. Furthermore, after the photo-polymerization process, the samples were pyrolyzed at 950 °C. The structure of the amorphous ceramics and the resulting ceramic yield and the decomposition behavior were investigated by FTIR spectroscopy and thermogravimetric analysis, respectively.

## 2. Materials and Methods

### 2.1. Materials

**Figure 1:**
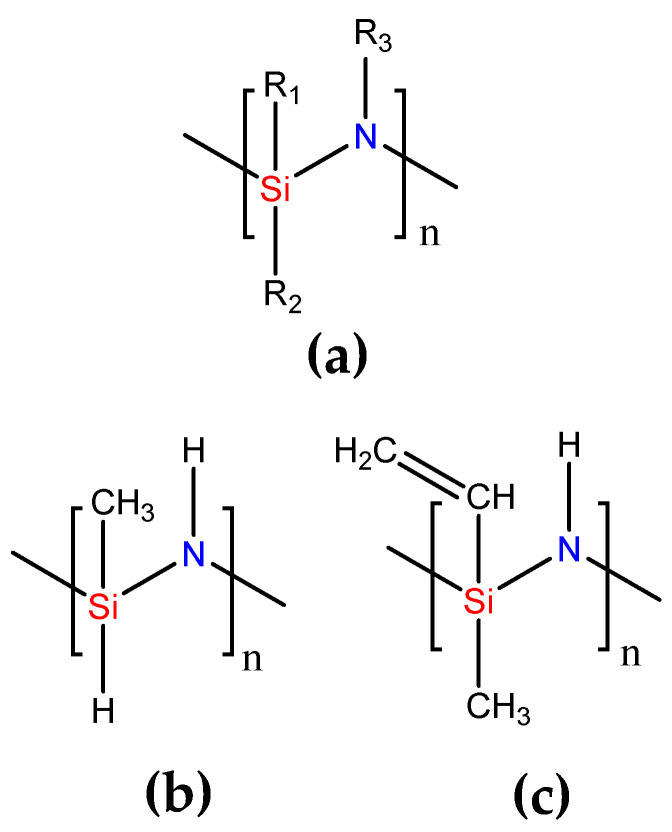
(**a**) Chemical structure of *Durazane 1800* with R_1_, R_2_, and R_3_, which are usually H, CH_3_, or CH=CH_2_ bonds, respectively, demonstrated in the chemical structures (**b**,**c**).

The compound utilized in this research is a commercially available liquid preceramic polymer, organopolysilazane (OPSZ, *Durazane 1800*, Merck KGaA, Darmstadt, Germany). The polymer consists of a silicon and nitrogen backbone, which is functionalized with different side groups, e.g., hydrogen, methyl, and vinyl groups. The simplified chemical structures are presented in Figure 1.

**Figure 2:**
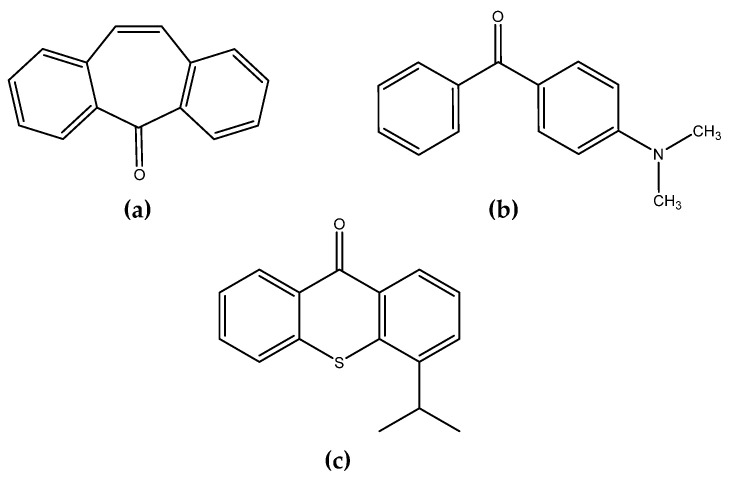
Photoinitiators used: (**a**) dibenzosuberonene (DBS), (**b**) 4-(dimethylamino)benzophenone (DMABP), and (**c**) isopropylthioxanthone (ITX).

Three different photoinitiators, dibenzosuberone (DBS, purity 98%, Merck KGaA, Darmstadt, Germany), 4-(dimethylamino)benzophenone (DMABP, purity 99%, Merck KGaA, Darmstadt, Germany), and isopropylthioxanthone (Genocure ITX, purity > 98.0%, Rahn AG, Zürich, Switzerland) (Figure 2), were added to *Durazane 1800* in order to investigate its curing behavior. All photoinitiators are derivates of benzophenone. 2-propanol (Carl Roth, 99.9%, Karlsruhe, Germany) was used as a solvent.

### 2.2. Preparation and Characterization

In a first series of experiments, the preceramic polymer *Durazane 1800* was mixed with three different photoinitiators to obtain photosensitive mixtures (see Table 1) and to investigate the polymerization behavior.

Each photoinitiator (1 wt% or 3 wt%) was dissolved in *Durazane 1800* (100 wt%) using a high shear disperser (T-10 basic Ultra-Turrax®, IKA, Germany) for 120 s at 9400 rpm. After the dissolution of the photoinitiator in *Durazane 1800*, the samples were irradiated for 300 s with LED light sources (LED-Spot-100 lamp, Dr. Hönle UV Technology, München, Germany) with different wavelengths (385 and 405 nm) in ambient (47% RH) or nitrogen atmosphere (1.1% RH). The relative humidity (RH) inside the UV chamber was measured by testo 608-H2 thermo hygrometer (Testo SE & Co. KGaA, Titisee-Neustadt, Germany). The intensity I_0_ of the UV lamps (λ = 385 or 405 nm) was 569 mW/cm^2^ and 553 mW/cm^2^, respectively. The intensity values were measured by the UV-Meter (HighEnd, Dr. Hönle UV Technology, Gräfelfing, Germany).

The Fourier-transform infrared (FT-IR) spectrometer was conducted to investigate the crosslinking behavior of the material. The spectra of all samples were recorded within a wavenumber range of 4000 and 500 cm^−1^ by averaging 32 scans at a resolution of 4 cm^−1^ using a Bio-Rad FTS 3000 Excalibur spectrometer (Varian, Palo Alto, CA, USA). The samples were coated with a doctor blade onto a shiny etched silicon wafer (~600 µm) and measured both uncured and UV-cured. The thickness of the samples on the silicon wafer was about 2 µm. A background measurement of a blank silicon wafer was taken first and substracted from each measured spectra.

The pyrolysis was performed in an alumina tube furnace (Carbolite, Neuhausen, Germany) at 950 °C, because the wafer is not resistant to higher temperatures. The heating rate was set at 1 °C/min and the temperature was held for 1 h at 950 °C using a nitrogen flow rate of 120 mL/min. Because of the different coefficient of thermal expansion of silicon compared with the resulting amorphous SiCN(O), the pyrolysis temperature was set to 950 °C to avoid cracks in the layer on the wafer.

The absorption spectra of the photoinitiators were measured by a UV-VIS spectrometer (Cary 500 Bio, Varian, Palo Alto, CA, USA) within a wavelength range of 450 and 220 nm. The photoinitiators were diluted in 2-propanol, filled into a quarz cuvette, and measured by dual-beam mode.

Thermogravimetric analysis (TGA) was performed using STA-409C (Netzsch Group GmbH &Co, Selb, Germany) to evaluate the polymer-to-ceramic conversion behavior and to examine the residual ceramic yield of all samples. Around 300 µL of each mixture was placed on a specimen and then the cured sample was scraped from the specimen. Around 20 mg of each cured sample was heated up to 1200 °C using a heating rate of 10 °C/min and a nitrogen flow rate of 100 mL/min. The experimental uncertainty for the residual mass measured by TGA is around ±1%.

### 2.3. Degree of Conversion of the Reactive Bonds in Durazane 1800

FTIR spectroscopy was used to determine the relative degree of conversion DC (%) of *Durazane 1800* and the photoinitiator after curing.

The degree of conversion was calculated using Equation (1) [32] for the chemical groups, which undergo chemical reactions during the crosslinking process: ≡Si–H, N–H, and the vinyl group.

(1) $$\mathrm{DC}\;\left(\%\right)=\left(1-\frac{A_{t}}{A_{0}}\right)\times 100$$

Thereby, the content of the integrated peak areas of the reactive bonds of the cured samples was defined as A_t_. The content of the reactive bond of the uncured sample, which was defined as A_0_, was taken as 100%. To minimize the influence of deviations in sample thickness and instrument recording, all integrals were normalized by the integral of the Si–CH_3_ bond at 1253 cm^−1^, as this bond does not change throughout the whole crosslinking process.

### 2.4. Crosslinking Mechanism of Polysilazane

Curing of preceramic polymers, such as *Durazane 1800*, usually takes place thermally by the addition of a free radical initiator like peroxides or catalysts. In this work, this approach was adopted, replacing the high temperature with the energy-efficient LED source to start the crosslinking reaction. Hence, as indicated before, three different photoinitiators were used for the polymerization of *Durazane 1800*.

**Figure 3:**
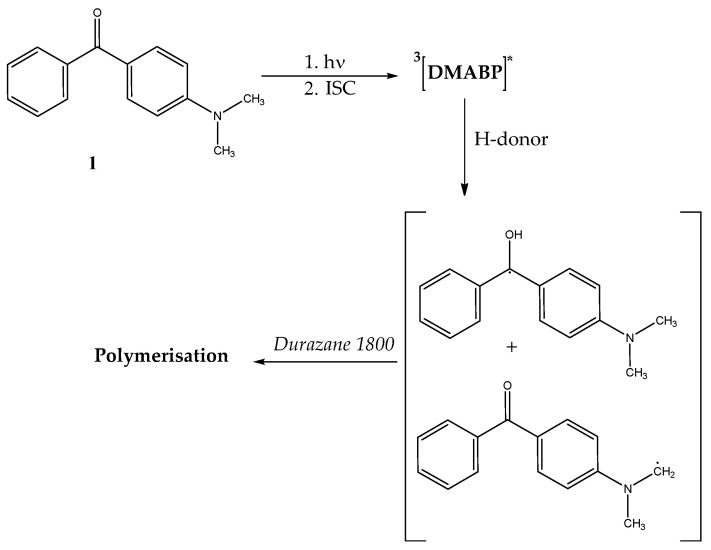
Schematic illustration of the initiation of DMABP upon UV light.

All used photoinitiators are derivates of benzophenone, which are Norrish type II photoinitiators [33], meaning that, when irradiated with UV light, they are excited to the singlet state, which subsequently changes to the triplet state via intersystem crossing (ISC) [34]. The mechanism is schematically illustrated in Figure 3 for the photoinitiator DMABP.

**Figure 4:**
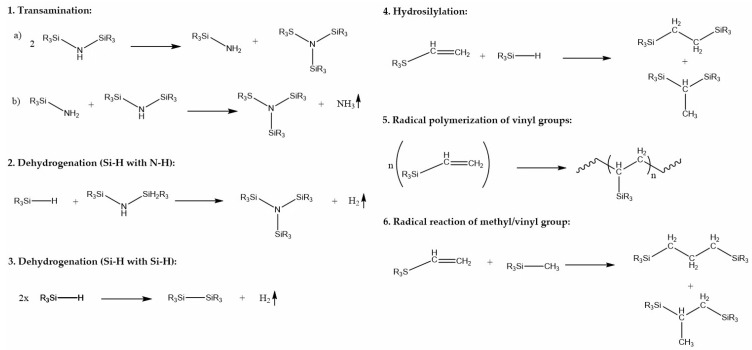
Several crosslinking reactions of *Durazane 1800* involve ≡Si-H, ≡Si–NH–Si≡, and RCH=CH2 groups that can occur in inert atmosphere [9].

In inert atmosphere, *Durazane 1800* can be primarily crosslinked through three different chemical bonds: ≡Si–H, ≡Si–NH–Si≡, and RCH=CH_2_ (vinyl group). The crosslinking reactions are shown in Figure 4.

The main group that undergoes a crosslinking reaction is the vinyl group. It is involved in the radical vinyl polymerization and hydrosilylation of Si-vinyl and ≡Si–H groups. The possible radical reaction of methyl and vinyl groups, which occurs at temperatures above 200 °C [35], can be neglected in this work. Moreover, the ≡Si–H bond undergoes dehydrogenation reactions between two ≡Si–H bonds and/or between ≡Si–H and ≡Si–NH–Si≡ groups. Finally, the ≡Si–NH–Si≡ group can crosslink via transamination reaction [7,9,36].

**Figure 5:**
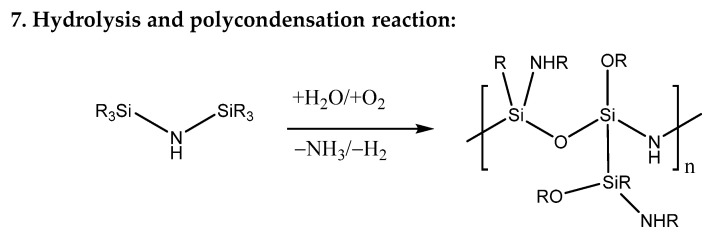
Hydrolysis and polycondensation reaction of *Durazane 1800* in ambient atmosphere.

In ambient atmosphere, the crosslinking reactions are mostly hydrolysis and polycondensation reactions [37]; these are shown in Figure 5. Thereby, the ≡Si–NH–Si≡ group reacts with a water or an oxygen molecule under formation of silanol groups, which subsequently polymerize to polysiloxane via polycondensation.

## 3. Results and Discussion

### 3.1. UV-VIS Spectroscopy

**Figure 6:**
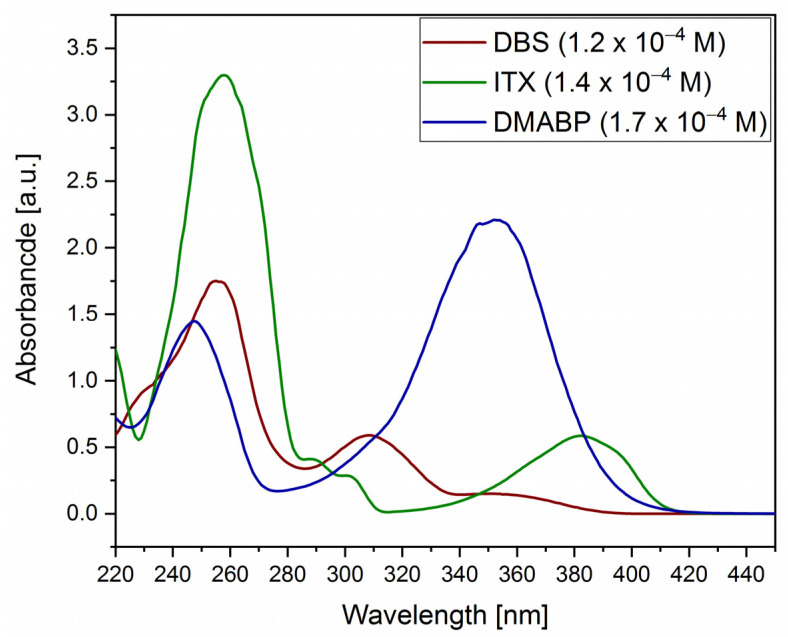
Absorption spectra of the photoinitiators in 2-propanol solution.

The UV-VIS spectra of the photoinitiators were recorded in order to distinguish at which absorption maximum the PIs absorb UV-light for starting the polymerization reaction of polysilazane. Figure 6 illustrates the absorbance spectra of the photoinitiators and Table 2 summarizes the absorption maxima.

The photoinitiator shows different absorption maxima, starting with ITX (Figure 6, green curve), which has two major absorption maxima at 258 nm and 382 nm in the UV-C and UV-A region, respectively, combined with two small shoulders between 290 and 301 nm in the UV-B region. The photoinitiator ITX is a derivate of benzophenone with a modification of a sulfur atom bridge between the two phenyl groups. Owing to the sulfur atom, the UV absorption spectra are redshifted compared with benzophenone [34].

As ITX, DBS (Figure 6) absorbs in the UV-region, showing the major maximum at 255 nm, followed by an absorption maximum at 307 nm and a small broad shoulder between 340 and 380 nm. Lastly, DMABP (Figure 6) is the only PI, which does not absorb in the UV-B region, showing its major absorption maximum in the UV-A region at 351 nm and the smaller absorption maximum at 248 nm. In this work, the UV-A region is of interest, as the employed light source has a light emitting diode for the emission of homogenous irradiation at a wavelength of 385 or 405 nm.

### 3.2. FTIR Analysis 

FTIR analysis was carried out before and after the UV curing to analyze the crosslinking behavior and to characterize the chemical structure of the samples. The relative degree of conversion was calculated for the reactive bonds (≡Si–H, ≡Si–NH–Si≡, and the vinyl group), which are detected by FTIR spectroscopy. The non-reactive group ≡Si–CH_3_, with a sharp band at about 1256 cm^−1^, is a common characteristic of all FTIR spectra of *Durazane 1800*, as it was synthesized through an ammonolysis reaction of dichloromethylsilanes (RCH_3_SiCl_2_, R=H, CH_3_, or CH=CH_2_) [7]. The ≡Si–CH_3_ group is used as a reference for all calculations of the crosslinking process.

**Figure 7:**
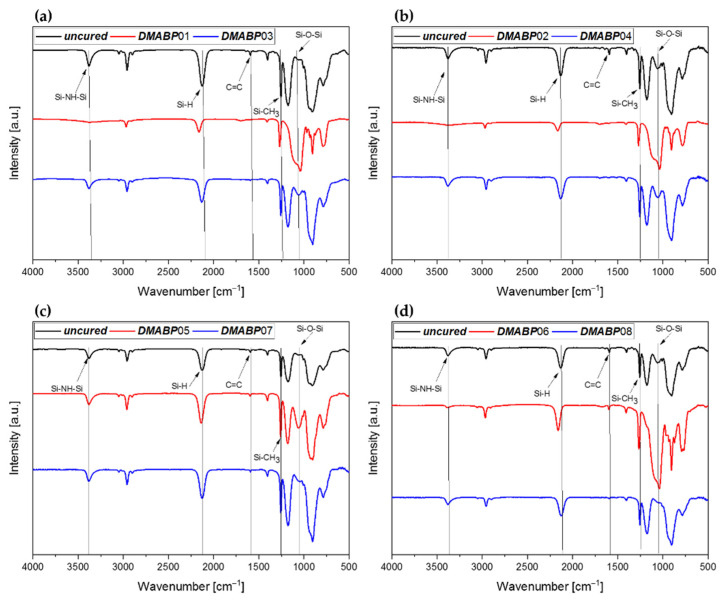
FITR spectra of the uncured and cured *Durazane 1800* sample mixed with DMABP as photoinitiator. The red curves are samples cured in ambient atmosphere and the blue curves in nitrogen atmosphere. Black curves belong to the uncured samples. (**a**) ***DMABP***01 and ***DMABP***03, (**b**) ***DMABP***02 and ***DMABP***04 cured by a UV lamp (λ = 385 nm). (**c**) ***DMABP***05 and ***DMABP***07, (**d**) ***DMABP***06 and ***DMABP***08 cured by a UV lamp (λ = 405 nm).

The FTIR spectra of ***DMABP***01–***DMABP***08 are presented in Figure 7a–d. The uncured samples (black curves) show the characteristic bands of pure *Durazane 1800*, which are mainly summarized in Table A1. The absorption bands below 1000 cm^−1^ relate to the stretching and deformation vibrations of Si–C, Si–N, C–C, and C–H bonds, which, however are overlapping and consequently cannot be accurately assigned.

In this work, the focus of attention lies on the reactive chemical bonds in the case of the uncured samples, including the vinyl group as well as the ≡Si–H and ≡Si–NH–Si≡ bond (Table 3). The absorption band at 1050 cm^−1^, corresponding to the ≡Si–O–Si≡, is only visible in Figure 7b,d. A possible reason for the ≡Si–O–Si≡ band in the uncured samples is the higher amount of photoinitiator, which might induce a slight silanol reaction during preparation in ambient atmosphere. After using *Durazane 1800*, the bottle is flooded with nitrogen to prevent oxygen contamination. Nevertheless, the oxygen cannot be completely avoided, as in the case of inert atmosphere.

**Figure 8:**
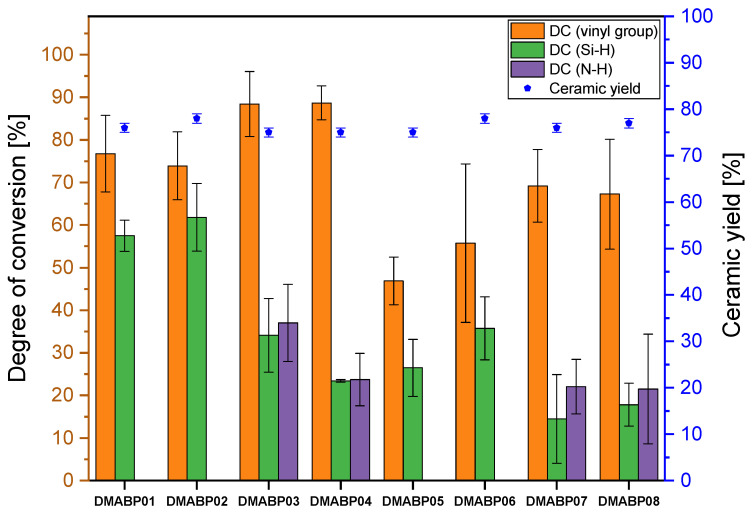
Degree of conversion (DC) of the reactive vinyl group as well as Si–H and N–H bonds calculated from the FTIR spectra of the samples ***DMABP***01–08. The ceramic yield was obtained by thermogravimetric analysis (TGA) measurement at 1200 °C.

The FTIR spectra of sample ***DMABP***03/04 and ***DMABP***07/08 were recorded after UV curing by a LED source (λ = 385 and 405 nm) in nitrogen atmosphere. In these conditions, the spectra of the cured samples reveal similar features as the uncured reference samples, except for the decrease of the deformation vibrations of the vinyl group (Figure 7, blue curves) at the wavenumber of 1596 cm^−1^. This indicates that the conversion of the vinyl group via radical polymerization is mainly responsible for the curing of *Durazane 1800*. The observation is supported by the calculation of the degree of conversion (DC, vinyl group) shown in Figure 8 and Table A1, which is three times as high as the DC of the Si–H and N–H bonds. The vinyl groups of samples ***DMABP***03/04 convert almost completely (DC = 89%) using the LED source (λ = 385 nm) compared with the samples ***DMABP***07/08. A reason for this occurence might be the absorption maximum of DMABP, which is at 351 nm (Table 2), being closer to 385 nm than to 405 nm. The DC (Si–H and N–H, Figure 8) of ***DMABP***03/04 is half as high as that of ***DMABP***01/02, owing to the absence of oxygen during the crosslinking process and, accordingly, little to no poly-condensation reactions of the Si–H and N–H groups occurred in inert atmosphere. The conversion of these bonds in inert atmosphere is based on transamination (N–H bond) and dehydrogenation (Si–H) reactions (see Figure 4). By comparing the spectra of ***DMABP***03/04 (blue curves) with ***DMABP***01/02 (red curves), the samples cured in an inert atmosphere exhibit a minor absorption band of ≡Si–O–Si≡ between 1080 and 1040 cm^−1^, which could be completely avoided when operating in a glovebox. Moreover, the photoinitiatior DMABP is resistant against oxygen inhibition, as the aminoalkyl radical can form a peroxide radical by reacting with oxygen. In turn the peroxide radical generates another aminoalkyl radical by hydrogen abstraction [34]. This property of DMABP can be very useful when operating with material suffering from oxygen inhibition. Hence, it can be concluded that successful curing of *Durazane 1800* took place using a low energetic LED source within minutes.

The FTIR spectra of ***DMABP***01/02 and ***DMABP***05/06 were measured after UV curing by an LED source (λ = 385 and 405 nm), respectively, in ambient atmosphere. For all samples cured in ambient atmosphere, the DC of the ≡Si–NH–Si≡ group was neglected, because the peak could not be distinguished properly owing to the peak broadening caused by water. The intensities of the bands assigned to the stretching of the C–H bonds of the vinyl group (at 3050 cm^−1^) as well as of the C=C double bonds at 1596 cm^−1^ disappeared for the samples ***DMABP***01/02 and decreased for the samples ***DMABP***05/06. Besides, the absorption band of the ≡Si–H bond decreased for all these samples. This observation indicates a hydrosilylation reaction (Figure 4, (4)) of the ≡Si–H group with the vinyl group and radical polymerization of the vinyl group (Figure 4, (5)). The disappearance of the vinyl group of the samples ***DMABP***01/02 may be due to the absorption maximum of the photoinitiator around 355 nm (Figure 6 and Table 2), thus corresponding more closely to the wavelength at 385 nm than to the 405 nm UV-lamp. This result is confirmed by the DC of the vinyl group, which is higher for ***DMABP***01/02 than for ***DMABP***05. However, it is in the same order of magnitude when comparing the samples ***DMABP***01/02 and ***DMABP***06, considering the measurement inaccuracy.

Moreover, the spectra of ***DMABP***01, ***DMABP***02, and ***DMAPB***06 (Figure 7a, Figure 7b, and Figure 7d, respectively, red curves) show the absorption band of ≡Si–O–Si≡. The reason is the hydrolysis of the ≡Si–NH–Si≡ group resulting in a silanol group (≡Si–OH) and ammonia, followed by a polycondensation reaction (Figure 5). This crosslinking reaction is confirmed by nearly complete disappearance of both the stretching and deformation vibrations of the Si–NH band. The absorption band of the Si–O–Si of sample ***DMABP***05 (Figure 7c) is not as strong in the case of ***DMABP***01/02 and ***DMABP***06, despite being polymerized similarly. In addition, neither the stretching nor the deformation vibrations of the ≡Si–NH–Si≡ group fully disappeared in this sample, despite curing in ambient atmosphere. One reason for this observation could be the low concentration of the photoinitiator compared with sample ***DMABP***06, and another reason could be the lower energetic irradiation of the LED source (λ = 405 nm). Moreover, the DC of the Si–H bond of samples ***DMABP***01/02 is higher than that of samples ***DMABP***05/06, implying more silanol and polycondensation reactions of the bonds ≡Si–H and N–H occurred at the wavelength 385 nm than at 405 nm. The hydrolysis of Si–H bond can be catalyzed by the ammonia formed from hydrolysis reactions of the ≡Si–NH–Si≡ group [39]. Especially in sample ***DMABP***05, the DC (≡Si–H) is much lower than in sample ***DMABP***06, indicating less hydrolysis and polycondensation reactions, as proven in the spectra (Figure 7c) by the unreacted absorption peaks of the ≡Si–NH–Si≡ bond. This result shows that *Durazane 1800* does not completely hydrolyse during curing, even in these conditions. Thermally, this could not be observed, as more energy is given into the system. However, this absorption band occurs for all samples cured in ambient atmosphere owing to the sensitivity of *Durazane 1800* to moisture [37,40,41].

Compared with DMABP and ITX, the samples mixed with DBS could not be cured by near visible light LED (λ = 405 nm), owing to its absorbance maxima (Figure 6) and the structure of DBS (Figure 2). As it only consists of phenyl-groups without heteroatom like sulfur or the amino alkyl group, the absorbance spectrum is not red-shifted. The photoinitiator concentration does not affect the DC (vinyl group, ≡Si–H, and N–H) for all samples. For the coating application, it is advantageous to use a higher amount of photoinitiator if thinner layers are desired [34].

**Figure 9:**
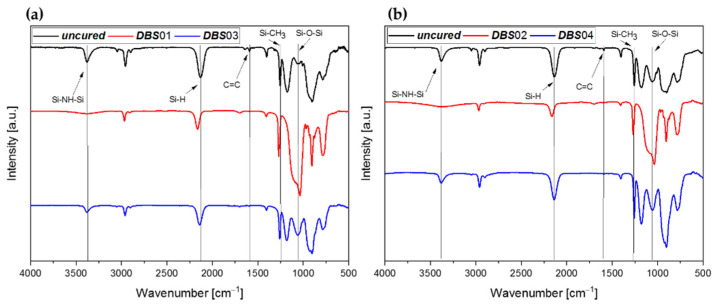
FTIR spectra of the uncured and cured *Durazane 1800* sample mixed with DBS as photoinitiator. The red curves were cured in ambient and the blue curves in nitrogen atmosphere. Black curves belong to the uncured sample. (**a**) ***DBS***01 and ***DBS***03, (**b**) ***DBS***02 and ***DBS***04 cured by the LED source (λ = 385 nm).

The samples ***DBS***01-04 were cured by the LED source (λ = 385 nm) in ambient and nitrogen atmosphere followed by the measurement of the FTIR spectra, which are illustrated in Figure 9a,b.

Samples ***DBS***03/04 were cured in nitrogen atmosphere and, as shown in Figure 9, an Si–O–Si absorption band (~1060 cm^−1^) was formed, implying that the samples were partially hydrolyzed. As shown in Figure 9b, the spectra of the uncured mixture already show an Si–O–Si band, indicating the sensitivity of the *Durazane 1800*/DBS system to oxygen. Nevertheless, the crosslinking behavior is supported by radical polymerization of the vinyl group (DC up to 85%), as the absorbance band of the vinyl group has decreased. Compared with ***DMABP***03/04, the DC (N–H) is higher for ***DBS***03/04 because the N–H group works as a co-initiator in the initiation reaction of DBS. Additionally, transamination and dehydrogenation reactions with the Si–H bond (DC ~ 40%) may have occurred.

**Figure 10:**
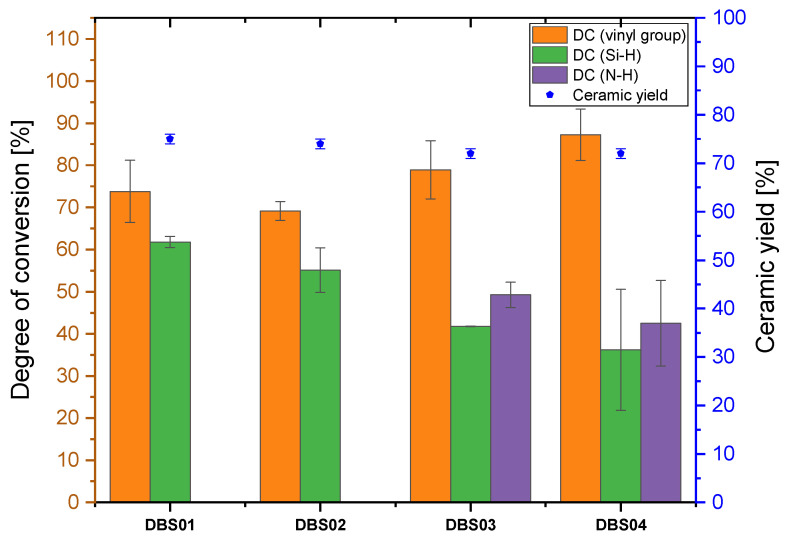
Degree of conversion of the reactive vinyl group as well as ≡Si–H and N–H bonds calculated from the FTIR spectra of samples ***DBS***01–04. The ceramic yield was obtained by TGA measurement at 1200 °C.

Similar to ***DMABP***01/02, samples ***DBS***01/02 cured in ambient atmosphere (red curves) also undergo hydrolysis and polycondensation reactions illustrated in Figure 9 by the formation of an Si-O-Si absorption band. Moreover, the vinyl group is converted as the absorption band decreases and the resulting DC (vinyl group) accounts for approximately 75% (Figure 10). The vinyl group possibly reacts with the Si-H bond (DC ~ 60%) by hydrosilylation reaction, forming an Si–C linkage, or via radical polymerization.

**Figure 11:**
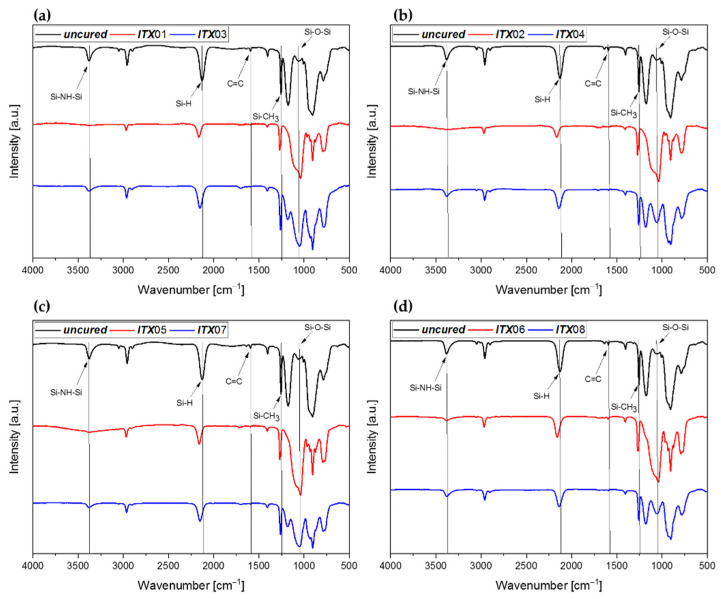
FTIR spectra of the uncured and cured *Durazane 1800* sample mixed with DMABP as photoinitiator. The red curves were cured in ambient atmosphere and the blue curves in nitrogen atmosphere. Black curves belong to the uncured sample. (**a**) ***ITX***01 and ***ITX***03, (**b**) ***ITX***02 and ***ITX***04 cured by the LED source (λ = 385 nm). (**c**) ***ITX***05 and ***ITX***07, (**d**) ***ITX***06 and ***ITX*** 08 cured by the LED source (λ = 405 nm).

**Figure 12:**
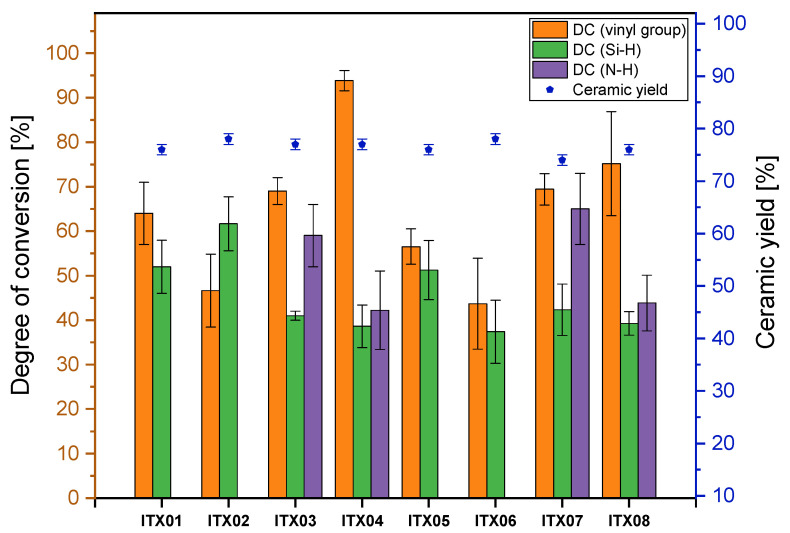
Degree of conversion of the reactive vinyl group as well as ≡Si–H and N–H bonds calculated from the FTIR spectra of the samples ***ITX***01–08. The DC (N-H) of the samples cured in nitrogen atmosphere. The ceramic yield was obtained by TGA measurement at 1200 °C.

By mixing *Durazane 1800* with the photoinitiator ITX, both LED sources could be used for curing the mixtures owing to the red shifting of ITX generated by the sulfur atom. The FTIR spectra and the resulting degree of conversion of the reactive bonds are shown in Figure 11 and Figure 12, respectively.

The initial aspect that is noticeable when analyzing the spectra of the samples (***ITX***03/04 and ***ITX***07/08) polymerized in nitrogen atmosphere is the formation of the ≡Si–O–Si≡ bond in spite of the inert atmosphere. The ≡Si–O–Si≡ bond is more evident for samples ***ITX***03/07 than for ***ITX***04/08. This result is represented by the corresponding DC (N-H), which is larger for ***ITX***03/07 than for ***ITX***04/08. One reason for this occurence might be the lower photoinitiator concentration, leading to a slower, nevertheless more homogenous, initiation of the photoinitiator, and thus to a slower gelation. However, as the N-H group co-initiates the photoinitiator, the conversion is higher with the lower concentrations of the initiator than at higher concentrations, because more radical centers could be activated owing to the slower reaction. The DC (vinyl group) of ***ITX***04 is higher than ***ITX***03, which is desirable, as the Si–C linkage observed by hydrosilylation reaction is stable at elevated temperatures.

As can be seen in the spectra (Figure 11), the samples (***ITX***01/02, ***ITX***05/06) cured in ambient atmosphere were hydrolyzed similarly to the aforementioned mixtures of *Durazane 1800* with DBS and DMABP. The calculated DC of the vinyl and the Si-H bonds are in the same order of magnitude for ***ITX***01/02 and ***ITX***05/06 and are to be attributed to hydrosilylation reactions and radical polymerization of the vinyl group.

**Figure 13:**
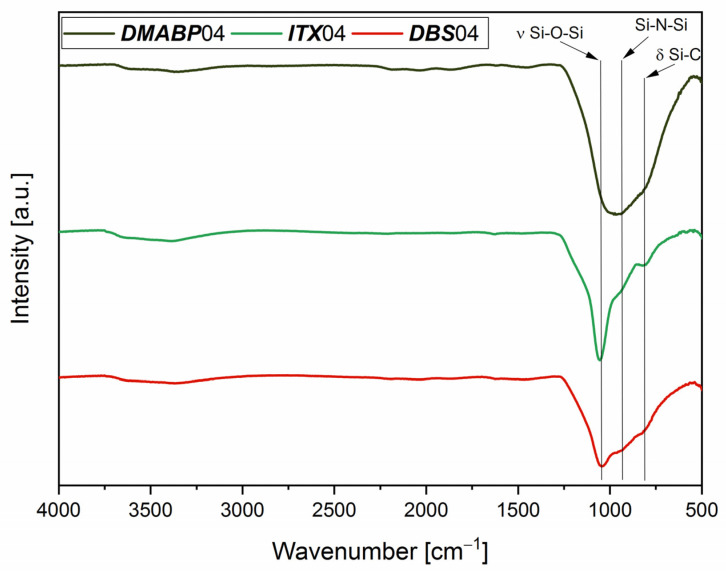
FTIR spectra of amorphous ***DMABP***04, ***ITX***04, and ***DBS***04 pyrolyzed in nitrogen at 950 °C.

The FTIR spectra of the samples pyrolyzed at 950 °C are shown in Figure 13 of the samples cured in nitrogen and ambient atmosphere. The ceramization process was completed as no absorption of Si–H or C–H bonds is present. A broad absorption band is visible between 1100 and 680 cm^−1^ attributed to Si–C, Si–N–Si, and Si–O–Si bonds. As previously mentioned, the presence of oxygen refers to the sensitivity of *Durazane 1800* to moisture. Even if the crosslinking and pyrolysis processes took place in nitrogen atmosphere, the mixtures were prepared in ambient atmosphere. As expected, the Si–O–Si bond is more evident for ***DBS***04 and ***ITX***04 compared with ***DMABP***04, as the spectra of the cured samples already showed the Si–O–Si absorption band.

### 3.3. Thermogravimetric Analysis and Ceramic Yields

**Figure 14:**
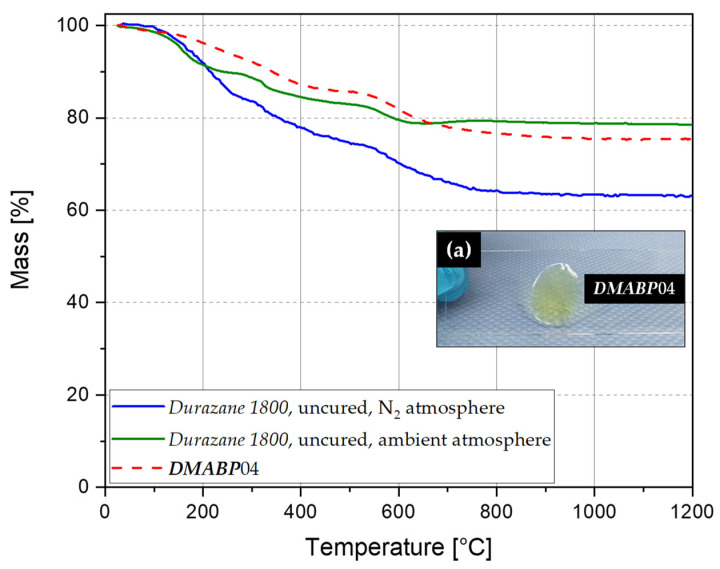
Thermograms of uncured *Durazane 1800* measured in ambient and nitrogen atmosphere (olive and blue curve). The thermogram of ***DMABP***04 was measured in nitrogen atmosphere after curing by an LED source (λ = 385 nm, N_2_). (a) Drop of ***DMABP***04 on a slide after UV curing.

**Figure A1:**
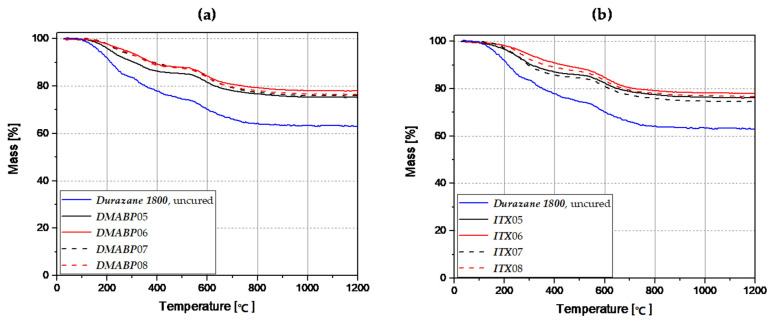
Thermograms of samples cured by an LED source (λ = 405 nm) measured in nitrogen atmosphere: (**a**) ***DMABP***05–08 and (**b**) ***ITX***05–08. The black curves are mixtures of *Durazane 1800* with 1 wt% photoinitiator concentration and the red curves are mixtures of *Durazane 1800* with 3 wt% PI concentrations. The dotted curves were cured in inert atmosphere and the non-dotted in ambient atmosphere. The blue curve belongs to pure uncured *Durazane 1800* measured in nitrogen atmosphere.

**Figure A2:**
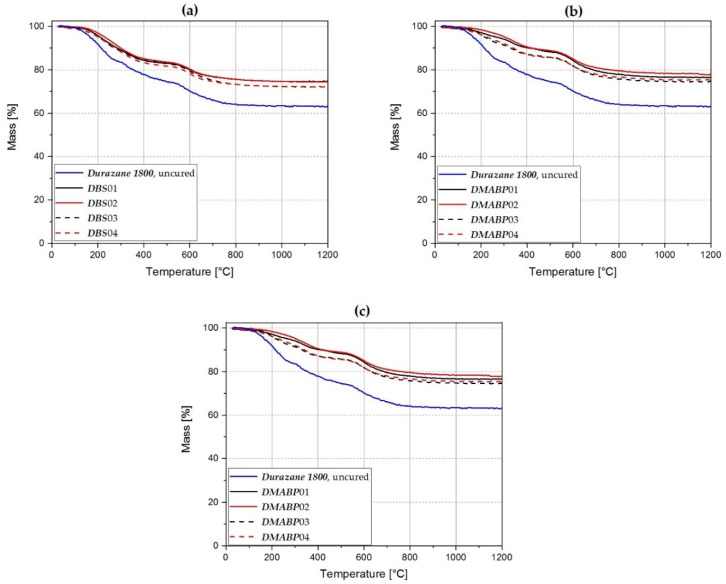
Thermograms of the samples cured by an LED source (λ = 385 nm) measured in nitrogen atmosphere: (**a**) ***DBS***01–04, (**b**) ***DMABP***01–04, and (**c**) ***ITX***01–04. The black curves are mixtures of *Durazane 1800* with 1 wt% photoinitiator concentration and the red curves are mixtures of *Durazane 1800* with 3 wt% PI concentration. The dotted curves were cured in inert atmosphere and the non-dotted in ambient atmosphere. The blue curve belongs to pure uncured *Durazane 1800* measured in nitrogen atmosphere.

Thermogravimetric analysis was conducted under nitrogen flow with 10 °C/min to study the decomposition of the preceramic polymer and to measure the ceramic yield of the sample. The ceramic yield of all samples was taken of the residual mass at 1200 °C and is plotted for each mixture in Figure 8, Figure 10, and Figure 12. The thermograms measured in nitrogen atmosphere are shown in Figure A1 and Figure A2 for the samples cured by the LED source (λ = 385 and 405 nm), respectively. Thermal behavior in nitrogen atmosphere will be explained using the sample ***DMABP***04 as an exemplary sample in comparison with uncured *Durazana 1800*, as the cured samples exhibit very similar thermal behavior. Sample ***DMABP***04 was cured by LED source (λ = 385 nm). The thermograms of uncured *Durazane 1800* measured in ambient and nitrogen atmosphere and of ***DMABP***04 are presented in Figure 14.

Pure and uncured *Durazane 1800*, which was measured both in nitrogen and ambient atmosphere, decomposes in a three-step process. The degradation starts below 100 °C with a mass loss of 16 and 10%, respectively, because of the non-crosslinked volatile oligomers. It is worth mentioning that the first step is interrupted by a small plateau between 200 and 300 °C, which is attributable to the free-radical polymerization of the vinyl groups. This plateau is missing in the UV-cured samples because the vinyl group was already radically converted. The second step begins between 300 and 530 °C and results in a mass loss of about 10 and 7%, respectively, owing to dehydrogenation and transamination reactions. During the third step, which starts between 530 and 850 °C, the polymer-to-ceramic transformation takes place as described before and results in a mass loss of 10 and 4%, respectively. The ceramic yield of pure *Durazane 1800*, measured in nitrogen and ambient atmosphere, is 63 and 80%, respectively. The main reason for the increase of ceramic yield is the incorporation of oxygen through hydrolysis and polycondensation reactions of *Durazane 1800* [40,42].

The degradation of the cured samples proceeds in a two-step process. The first step starts at temperatures between 120 and 400 °C. During this step, further dehydrogenation and transamination reactions occur, leading to mass loss due to volatile groups like ammonia and hydrogen. Furthermore, at temperatures higher than 100 °C, the non-crosslinked volatile oligomers degrade, resulting in mass loss. On the other hand, at temperatures higher than 200 °C, the residual vinyl groups undergo further radical polymerization and form a thermoset. Therefore, the curve between 200 and 400 °C is more flat than sharp. The second step starts between 500 and 750 °C, resulting in a mass loss due to the organic–inorganic transformation of the thermoset into amorphous SiCN and SiCO, depending on the curing atmosphere. The organic substituents degrade into methane and other volatile hydrocarbons, hydrogen, ammonia, and volatile silicon derivatives [12], mainly owing to rearrangements and radical reactions leading to bond breaking and new bond formation [43]. No further mass loss is observed at temperatures higher than 800 °C.

In comparison with the uncured *Durazane 1800* (63% in N_2_ atmosphere), the ceramic yield of the samples crosslinked via LED source (λ = 385 and 405 nm) increased significantly up to the range of between 72 and 78% depending on the mixture and crosslinking atmosphere (see Appendix A, Table A1). Crosslinking of the preceramic polymers is necessary for the polymer-to-ceramic transformation, as it increases the ceramic yield by reducing the volatilization of the oligomers [44]. The high ceramic yield confirmed the high degree of conversion. In addition, it demonstrates the response of *Durazane 1800* to the chosen crosslinking approach. For increasing the ceramic yield and reducing the shrinkage of the final ceramic, crosslinking is an indispensable process, as the uncured sample is at least 10% lower than the crosslinked samples in inert atmosphere.

In this work, different parameters were changed to examine the behavior of *Durazane 1800*. First, the influence of the polymerization atmosphere on the ceramic yield was studied. As summarized in Table A1, the ceramic yield of ***DBS***01/02 is slightly higher than that of ***DBS***03/04, owing to the already mentioned oxygen incorporation, which is visible in the FTIR spectra (Figure 9). A higher ceramic yield was also observed for ***DMABP***02 in comparison with ***DMABP***03/04. For all the other samples, the polymerization atmosphere did not affect the ceramic yield. Furthermore, the photoinitiator concentration was varied for the purpose of examining the influence on the ceramic yield. The higher photoinitiator concentration does not affect the ceramic yield for most samples, except for sample ITX08, which has a higher ceramic yield than ITX07. Finally, two UV-lamps with different wavelengths were used for the photoinitiators ITX and DMABP. The change of the LED source did not influence the ceramic yield, because the ceramic yield depends on the conversion of the reactive bonds. As previously described, the mixtures presented in Table 1 were cured properly by the chosen conditions and parameter.

## 4. Conclusions

In conclusion, the present work focuses on the crosslinking behavior of polyorganosilazane (*Durazane 1800*), which was mixed with three different photoinitiators—dibenzosuberone (DBS), 4-(dimethylamino)benzophenone (DMABP), and isopropylthioxanthone (ITX)—and UV irradiated by two LED sources (λ = 385 and 405 nm) in ambient and nitrogen atmosphere. It can be concluded that successful curing of *Durazane 1800* was established within minutes using energy-efficient LED sources. The crosslinking behavior of the mixtures was investigated in detail by FTIR spectroscopy and the degree of conversion was calculated for the reactive bonds. The curing atmosphere has a significant impact on the crosslinking behavior of the reactive bonds and the chemical composition of the resulting ceramic. In nitrogen atmosphere, it turned out that the DMABP mixtures observed the least oxygen incorporation in comparison with the other two photoinitiators. The main reason for this occurence is the aminoalkyl radical, which can form a peroxide radical by reacting with oxygen. The peroxide radical in turn generates another aminoalkyl radical by hydrogen abstraction [34]. The resulting ceramic of the samples cured in nitrogen atmosphere is SiCN(O). The amount of oxygen depends on the curing kinetics of the photoinitiator. If the reactivity of the photoinitiator is slow, the N–H bond will hydrolyse beforehand. Therefore, DMABP is recommended for usage as it shows the best results in terms of insensitivity to hydrolysis reactions.

The approach presented in this work can be applied to various applications, including coatings’ or microelectromechanical systems’ (MEMS) fabrication. Especially in the field of MEMS, the preparation of precise structures is required, so it is of great interest if a targeted curing can be carried out by means of a mask, which would not be possible with thermal curing. With the help of this approach, UV-curable inks for inkjet printing can be prepared or even 3D structures can be fabricated by stereolithography.

In ambient atmosphere, curing of all samples is given by partial hydrolysis and polycondensation reactions owing to the sensistivity of poly(organo)silazane to water. Therefore, the resulting ceramic will be SiCO(N).

As only one spectroscopic method (FTIR) was applied in this work, it is of great importance that other methods are used to better understand and compare the presented system. FTIR spectroscopy is a fast and easy to use method. However, one disadvantage of the technique is the overlapping of the bands, which makes the method imprecise. Therefore, the use of another method such as NMR spectroscopy is indispensable, as it would identify individual bonds quite accurately.

By comparing the two used LED sources (λ = 385 and 405 nm) of the DMABP samples, it was found that the samples cured by the LED source (λ = 385 nm) reached the higher degree of conversion of the reactive bonds, because this wavelength corresponds more closely to the absorption maximum of the photoinitiator. When increasing the photoinitiator concentration, a high radical concentration is available near the surface, resulting in sufficient surface cure. Accordingly, the thickness of the sample needs to be reduced to achieve complete curing as most of the light is absorbed on the surface, leading to a top to bottom amount of initiated species [45]. A lower photoinitiator concentration causes a homogeneous radical distribution, leading to good through-curing, but poor surface cure [34]. The photoinitiator concentration should be adjusted for each application depending on the film thickness desired. Moreover, the higher amount of photoinitiator results in a faster gelation owing to the higher radical concentration.

The ceramic yield was obtained by using the residual mass of the TGA and was observed in the range of 72 to 78% depending on the composition and crosslinking behavior. Hence, the curing atmosphere only influenced the ceramic yield of the DBS mixtures. In addition, the two different LED sources and the photoinitiator concentration do not affect the ceramic yield. Thus, the ceramic yield observed by the approach presented is high enough to form dense amorphous ceramics.

## Figures and Tables

**Table 1:** Overview of the sample description with respect to the used UV lamp and atmosphere as parameters for the polymerization process. All mixtures contain 100 wt% *Durazane 1800* and were cured for 300 s.

				Polymerization	
Sample	Initiator	c (Initiator)	c_i_ (Initiator)	UV Lamp	Atmosphere
		[wt %]	(mol/L)	[nm]	
***DBS***01	DBS	1	1.6 × 10^−2^	385	Ambient
***DBS***02	DBS	3	4.8 × 10^−2^	385	Ambient
***DBS***03	DBS	1	1.6 × 10^−2^	385	N_2_
***DBS***04	DBS	3	4.8 × 10^−2^	385	N_2_
***DMABP***01	DMABP	1	1.5 × 10^−2^	385	Ambient
***DMABP***02	DMABP	3	4.4 × 10^−2^	385	Ambient
***DMABP***03	DMABP	1	1.5 × 10^−2^	385	N_2_
***DMABP***04	DMABP	3	4.4 × 10^−2^	385	N_2_
***DMABP***05	DMABP	1	1.5 × 10^−2^	405	Ambient
***DMABP***06	DMABP	3	4.4 × 10^−2^	405	Ambient
***DMABP***07	DMABP	1	1.5 × 10^−2^	405	N_2_
***DMABP***08	DMABP	3	4.4 × 10^−2^	405	N_2_
***ITX***01	ITX	1	1.3 × 10^−2^	385	Ambient
***ITX***02	ITX	3	3.9 × 10^−2^	385	Ambient
***ITX***03	ITX	1	1.3 × 10^−2^	385	N_2_
***ITX***04	ITX	3	3.9 × 10^−2^	385	N_2_
***ITX***05	ITX	1	1.3 × 10^−2^	405	Ambient
***ITX***06	ITX	3	3.9 × 10^−2^	405	Ambient
***ITX***07	ITX	1	1.3 × 10^−2^	405	N_2_
***ITX***08	ITX	3	3.9 × 10^−2^	405	N_2_

**Table 2:** Summary of the absorption maxima of the photoinitators classified according to the different UV ranges.

	UV-C	UV-B	UV-A
Photoinitiator	Absorption Maximum λ_max_	Absorption Maximum λ_max_	Absorption Maximum λ_max_
	[nm]	[nm]	[nm]
DBS	255	307	352
ITX	258	290; 301	382
DMABP	248	-	351

**Table 3:** Overview of important FTIR stretching and deformation vibrations in *Durazane 1800* [38].

Chemical Bonds	Stretching Vibrations	Deformation Vibrations
	[cm^−1^]	[cm^−1^]
≡Si–NH–Si≡	3382	1176
C=C double bond in vinyl group		1596; 1400
≡Si–CH_3_		1257
≡Si–CH_2_–CH_2_–Si≡	1180–1120	
≡Si–O–Si≡	1080–1040	

**Figure A3:**
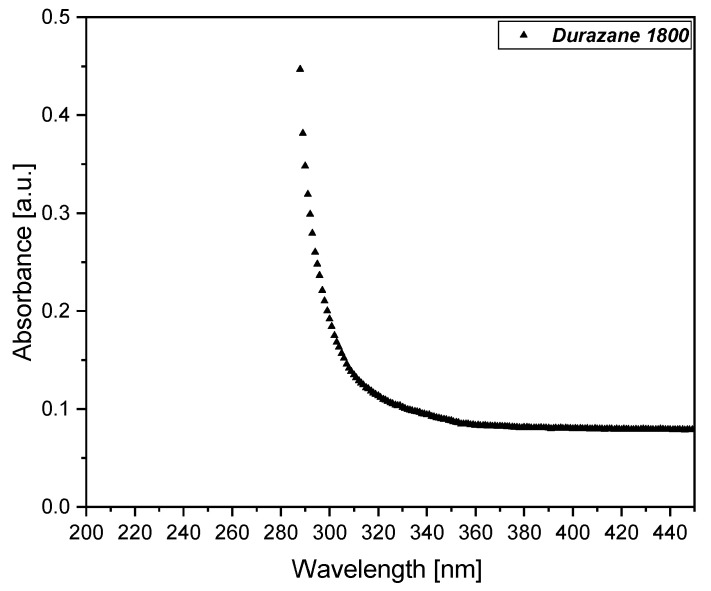
UV-VIS spectra of pure *Durazane 1800*.

## Data Availability

Not applicable.

## Appendix A

**Table A1:** Ceramic yield of the UV-cured samples measured from the residual mass of TGA at 1200 °C. The degree of conversion (DC) of the reactive samples calculated by the absorption bands of the FTIR spectra.

Sample	DC (Vinyl Group)	DC (≡Si–H)	DC (N–H)	Ceramic Yield at 1200 °C
	[%]	[%]	[%]	[%]
***Durazane 1800*** uncured, N_2_ atmospere	-	-	-	63
***Durazane 1800*** uncured, ambient atmospere	-	-	-	80
***DBS***01	74 ± 7	62 ± 1	-	75
***DBS***02	69 ± 2	55 ± 5	-	74
***DBS***03	79 ± 7	41.8 ± 0.1	49 ± 3	72
***DBS***04	87 ± 6	36 ± 14	43 ± 10	73
***DMABP***01	77 ± 9	57 ± 4	-	77
***DMABP***02	74 ± 8	62 ± 8	-	78
***DMABP***03	88 ± 8	34 ± 9	37 ± 9	75
***DMABP***04	89 ± 4	23.4 ± 0.3	24 ± 6	76
***DMABP***05	47 ± 6	26 ± 7	-	75
***DMABP***06	56 ± 19	36 ± 7	-	78
***DMABP***07	69 ± 9	14 ± 10	22 ± 6	76
***DMABP***08	67 ± 13	18 ± 5	22 ± 13	77
***ITX***01	64 ± 7	52 ± 6	-	76
***ITX***02	47 ± 8	61 ± 6	-	78
***ITX***03	69 ± 3	41 ± 1	59 ± 7	77
***ITX***04	94 ± 2	39 ± 5	42 ± 9	77
***ITX***05	57 ± 4	51 ± 7	-	76
***ITX***06	44 ± 10	37 ± 7	-	78
***ITX***07	69 ± 4	42 ± 6	65 ± 8	74
***ITX***08	75 ± 12	39 ± 3	44 ± 6	76

**Table A2:** Overview of FTIR stretching and deformation vibrations in *Durazane 1800* [38].

Chemical Bonds	Stretching Vibrations	Deformation Vibrations
	[cm^−1^]	[cm^−1^]
N–H	3382	1176
C–H (vinyl)	3046	
C–H in CH_x_ (CH_3_ asymmetric)	2957	
C–H in CH_x_ (CH_2_ asymmetric)	2902	
C–H in CH_x_ (CH asymmetric)	2804	
≡Si–H	2135	
C=C double bond in vinyl group		1596
CH_3_		1400
≡Si–CH_3_		1257
≡Si–CH_2_–CH_2_–Si≡	1180–1120	
≡Si–O–S≡	1080–1040	
≡Si–N–S≡		
≡Si–CH=CH_2_	950	
